# Implementing Technologies to Enhance Coordinated Specialty Care Framework: Implementation Outcomes From a Development and Usability Study

**DOI:** 10.2196/46491

**Published:** 2023-10-03

**Authors:** James B Green, Joey Rodriguez, Matcheri Keshavan, Paulo Lizano, John Torous

**Affiliations:** 1 Beth Israel Deaconess Medical Center, Harvard Medical School Boston, MA United States; 2 Brookline Center for Community Mental Health Brookline, MA United States

**Keywords:** psychosis, digital health, digital mental health, coordinated specialty care, digital navigator, clinical high risk, schizophrenia, implementation science, technology, mobile phone

## Abstract

**Background:**

Coordinated specialty care (CSC) has demonstrated efficacy in improving outcomes in individuals at clinical high risk for psychosis and individuals with first-episode psychosis. Given the limitations of scalability and staffing needs, the augmentation of services using digital mental health interventions (DMHIs) may be explored to help support CSC service delivery.

**Objective:**

In this study, we aimed to understand the methods to implement and support technology in routine CSC and offered insights from a quality improvement study assessing the implementation outcomes of DMHIs in CSC.

**Methods:**

Patients and clinicians including psychiatrists, therapists, and supported education and employment specialists from a clinical-high-risk-for-psychosis clinic (Center for Early Detection Assessment and Response to Risk [CEDAR]) and a first-episode–psychosis clinic (Advancing Services for Psychosis Integration and Recovery [ASPIRE]) participated in a quality improvement project exploring the feasibility of DMHIs following the Access, Alignment, Connection, Care, and Scalability framework to implement mindLAMP, a flexible and evidenced-based DMHI. Digital navigators were used at each site to assist clinicians and patients in implementing mindLAMP. To explore the differences in implementation outcomes associated with the app format, a menu-style format was delivered at CEDAR, and a modular approach was used at ASPIRE. Qualitative baseline and follow-up data were collected to assess the specific implementation outcomes.

**Results:**

In total, 5 patients (ASPIRE: n=3, 60%; CEDAR: n=2, 40%) were included: 3 (60%) White individuals, 2 (40%) male and 2 (40%) female patients, and 1 (20%) transgender man, with a mean age of 19.6 (SD 2.05) years. Implementation outcome data revealed that patients and clinicians demonstrated high accessibility, acceptability, interest, and belief in the sustainability of DMHIs. Clinicians and patients presented a wide range of interest in unique use cases of DMHI in CSC and expressed variable feasibility and appropriateness associated with nuanced barriers and needs. In addition, the results suggest that adoption, penetration, feasibility, and appropriateness outcomes were moderate and might continue to be explored and targeted.

**Conclusions:**

Implementation outcomes from this project suggest the need for a patient- and clinician-centered approach that is guided by digital navigators and provides versatility, autonomy, and structure. Leveraging these insights has the potential to build on growing research regarding the need for versatility, autonomy, digital navigator support, and structured applications. We anticipate that by continuing to research and improve implementation barriers impeding the adoption and penetration of DMHIs in CSC, accessibility and uptake of DMHIs will improve, therefore connecting patients to the demonstrated benefits of technology-augmented care.

## Introduction

### Background

Coordinated specialty care (CSC) is the gold standard of care for the treatment of individuals at clinical high risk for psychosis (CHR-p) and with first-episode psychosis (FEP) and has demonstrated efficacy in reducing hospitalization visits and improving symptoms and functioning. CSC teams generally include (1) team leadership, (2) case management, (3) supported employment and education, (4) psychotherapy, (5) family education and support, and (6) pharmacotherapy and primary care coordination [1]. However, this also makes the CSC program staff intensive and limits their scalability. Recent research has suggested that CSC care delivery may be supported through digital mental health interventions (DMHIs) in FEP care [2] and CHR-p care [3]; however, its feasibility and effectiveness are yet to be demonstrated.

Patients with FEP have high rates of technology ownership and use as evidenced by a 2022 study of an FEP clinic that found that patients’ access to smartphones was 100% and that 66% of the patients used social media [4]. A variety of new technologies have been studied in CSC including apps, web-based videos, games, and social media platforms [5]. Although these studies had smaller sample sizes, they show high levels of interest from patients. However, more formal studies of technology and apps for CSC have shown more disappointing results likely due to low engagement [6,7] that may be related to implementation challenges such as acceptability and usability for participants [8].

However, low engagement and uptake need not be barriers. These are common challenges across all digital health platforms, meaning that there are common solutions. Proposed solutions beyond patient-centered design include ensuring that technology use is customized to patient needs and that clinicians have the support they need to implement and troubleshoot the technology. An ongoing study in an FEP program in Spain [9] demonstrated that when apps are integrated into care and are used in conjunction with a clinician, engagement can be high. Integrating apps in this manner requires consideration of CSC-specific challenges and thinking beyond just the technology. To address implementation challenges, implementation science offers guidelines to support (1) the identification of barriers and facilitators across different implementation targets and (2) the development of an implementation strategy to address barriers and enhance facilitators [10].

Although there are several implementation frameworks to guide efforts, one practical model to apply is the Access, Alignment, Connection, Care, and Scalability (AACCS) [11] framework, which uses digital navigators to measure and intervene upon specific implementation facilitators and barriers of technology in a CSC setting.

Given that recent meta-analyses show promise that digital technologies can effectively enhance care in psychosis treatment [12], it is of significance to explore the effectiveness of digital technologies on symptomatic and functional outcomes in concert with implementation outcomes. Assessing these outcomes in concert may shed light on the necessary next steps needed to bring the benefit of digital technologies to the clinical setting.

This quality improvement project aims to develop and use a novel protocol, Implementing Technologies to Enhance Coordinated Specialty Care (iTECSC), to assess the implementation outcomes for digital technologies in CSC. iTECSC is a hybrid type-2 [13] protocol intended to analyze implementation outcomes and the effectiveness of digitally supported treatment in a CSC setting. Guided by implementation science, the outlined protocol was informed by the AACCS framework and supported by a small quality improvement study at 2 local CSC clinics that attempted to identify, measure, and intervene upon implementation barriers and facilitators.

### Development of iTECSC

#### Overview

The implementation blueprint and outcomes facet of the protocol were developed by drawing on cutting-edge suggestions in the digital technology literature and by conducting a small quality improvement project at 2 local CSC clinics.

By integrating the AACCS implementation framework [11], implementation outcomes [14], suggestions from implementation science [10], and recent studies analyzing the importance of digital navigators [15], we designed a multistage blueprint to assist the evaluation of implementation outcomes.

#### AACCS Framework

The AACCS framework is a 5-stage implementation model that this study used as a guide to implement DMHIs. This framework uses a bottom-up approach to address implementation needs in community settings starting with *access* to technology, *alignment* of technology to support clinical needs, *connection* of the technology to those needs, ongoing *care* to support the implementation of technology into treatment, and supporting the *sustainability* of the technology.

**Figure 1:**
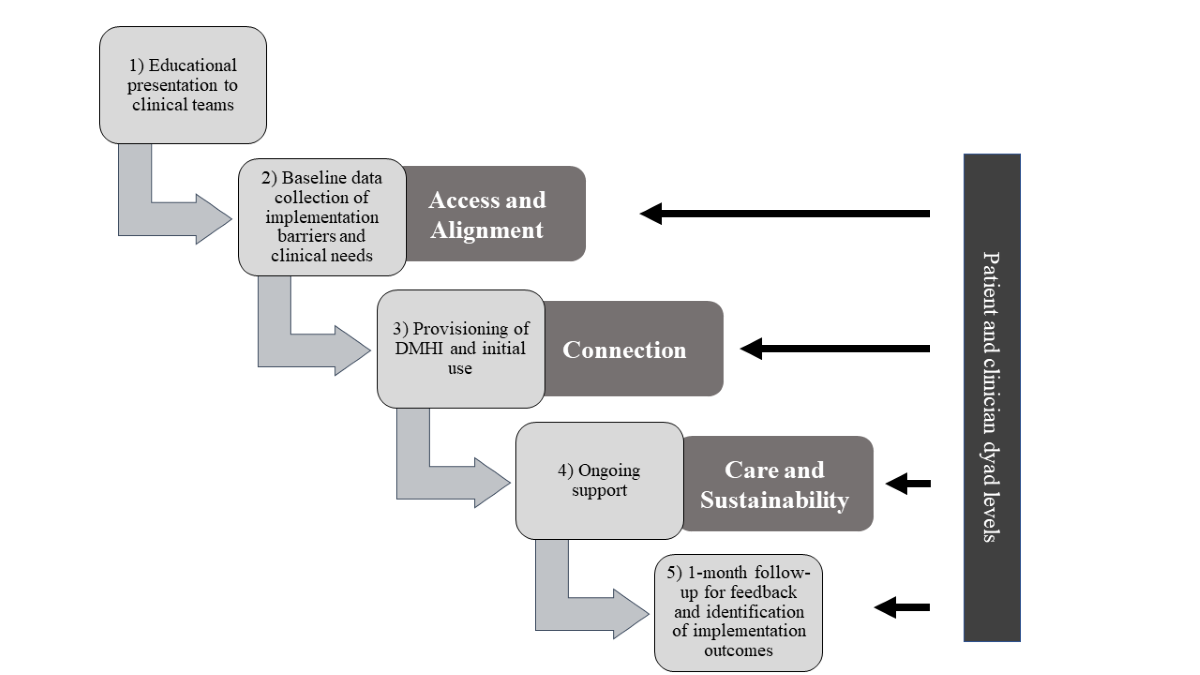
Implementation design using the Access, Alignment, Connection, Care, and Scalability (AACCS) framework. DMHI: digital mental health intervention.

Following the AACCS framework, this project developed practical steps to address each need. These steps included (1) an initial educational presentation to the clinical teams about the DMHI opportunity, (2) baseline data collection to identify implementation barriers and inform DMHI development, (3) provisioning of the DMHI and hands-on support to clinicians and patients with initial use, (4) providing ongoing support to resolve questions or adjust the DMHI to patient-specific needs, and (5) collection of 1-month follow-up data to collect general feedback and identify specific implementation barriers and facilitators (Figure 1). Steps 2 to 5 targeted both clinician and patient dyads, and all steps were supported by trained digital navigators.

#### Digital Navigators

Digital navigators [15] are staff trained to be able to support digital health in a clinical context. They do not need to be clinicians but must have gone through specialized training around supporting Apple and Android smartphones, app evaluation, clinical safety, data evaluation, and digital engagement per published curriculum. Digital navigators are trained to offer flexible support and can be deployed in diverse clinical settings as illustrated in these 5 cases. This protocol uses digital navigators at all stages of implementation.

#### MindLAMP

**Figure 2:**
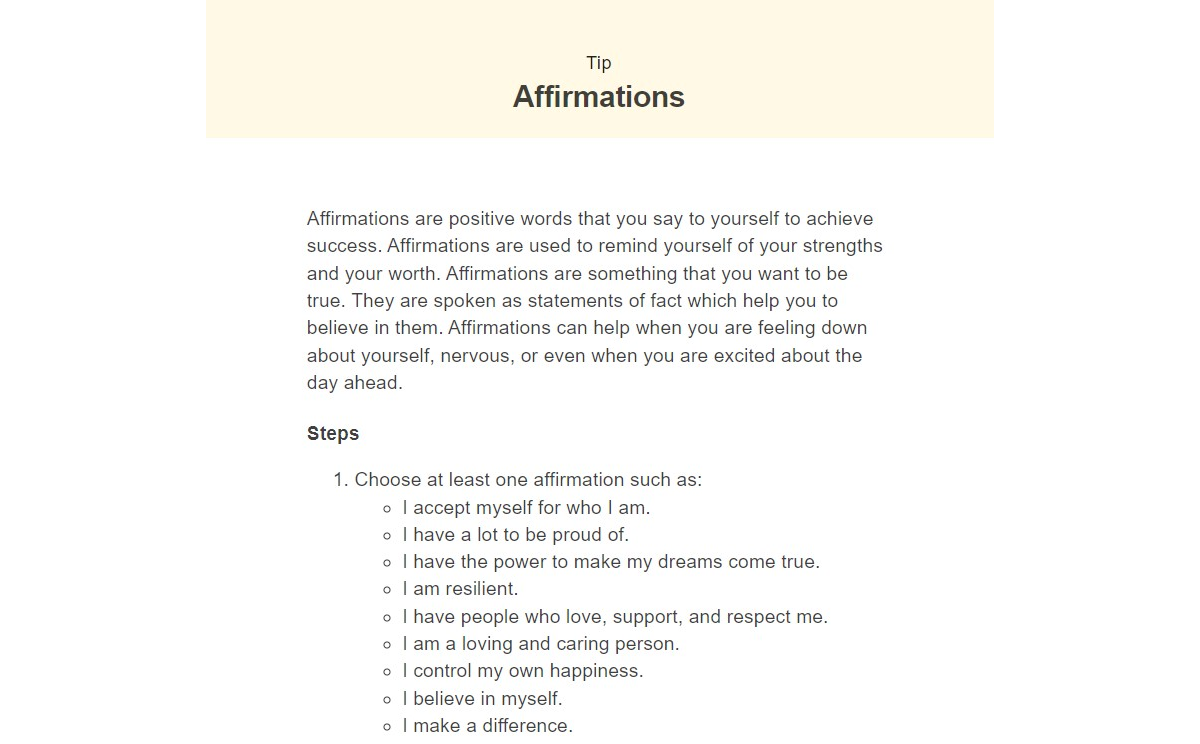
Learn with affirmations.

**Figure 3:**
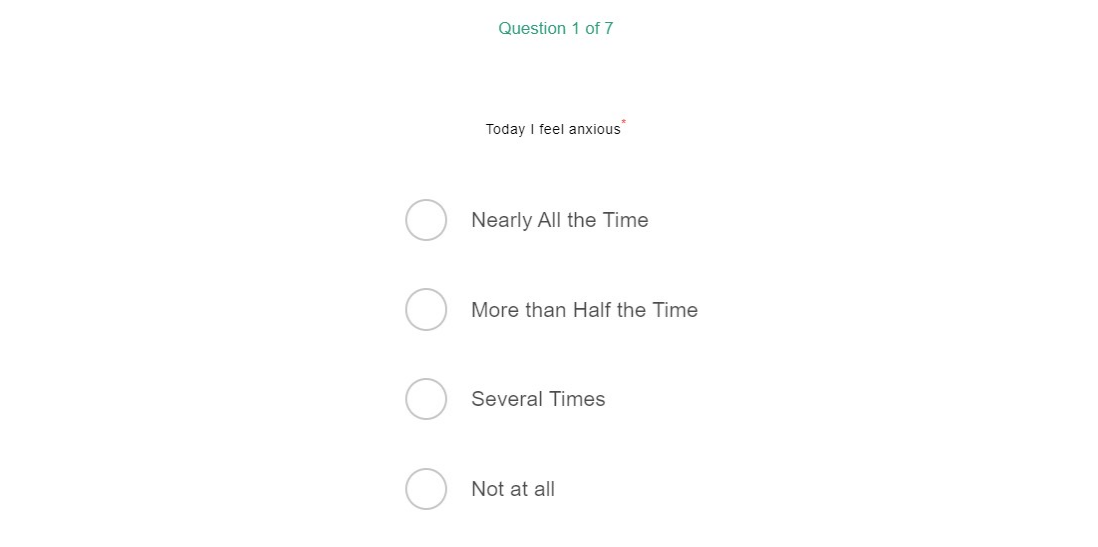
Assess with questionnaires.

**Figure 4:**
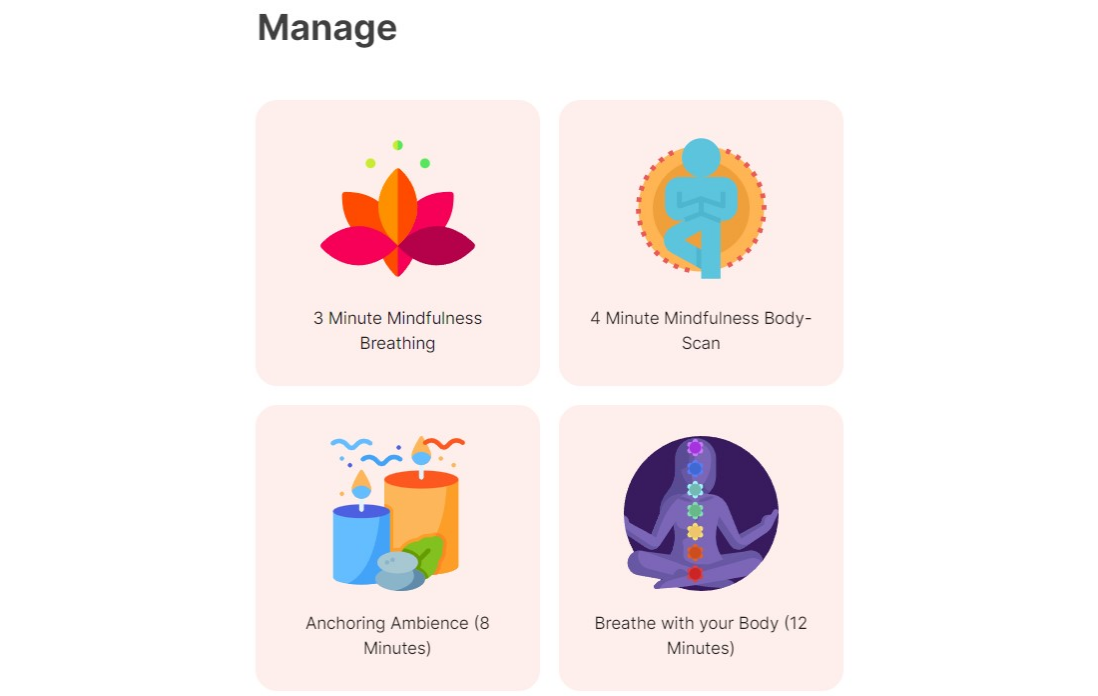
Manage with activities.

**Figure 5:**
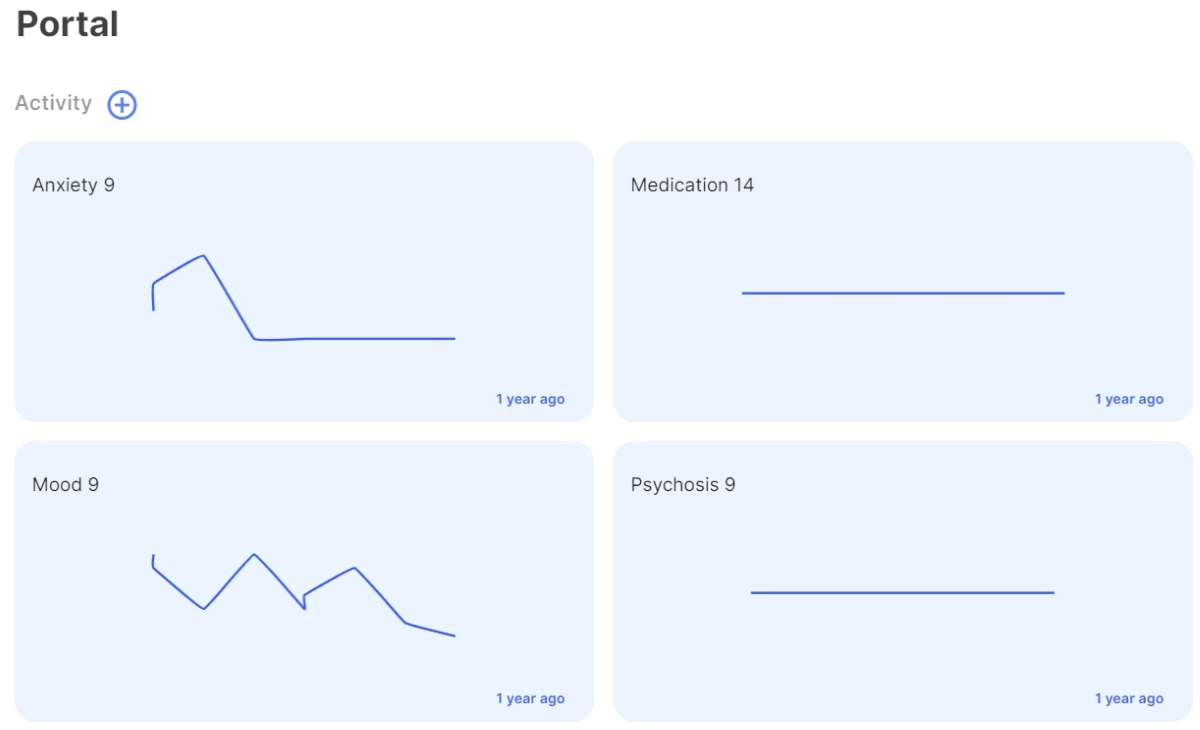
Prevent with measurement-based care.

MindLAMP is a flexible Apple and Android smartphone app co-developed by people with lived experience and freely shared as an open-source software [16]. The mindLAMP app was the platform in which the DMHI was delivered in this protocol. It is unique because the entire app can be customized to the needs of any study, clinic, or patient group. The *Learn*, *Assess*, *Manage*, and *Prevent* tabs of mindLAMP allow for custom psychoeducation, surveys, activities, feedback, and measurement-based care to be shared between patients and clinicians. This offers the advantage of not needing to create a new app for each new use case and an adaptable and versatile platform to meet patient-specific needs. Example use cases of the Learn, Assess, Manage, and Prevent features are shown in Figures 2-5.

#### Implementation Science and Implementation Outcomes

Implementation outcomes were used in the quality improvement project to assess for barriers and facilitators of implementation: (1) acceptability, (2) adoption, (3) appropriateness, (4) cost, (5) feasibility, (6) fidelity, (7) penetration, and (8) sustainability [14]. As implementation science suggests that barriers and facilitators may exist on multiple levels of context, this protocol targets both clinicians and patients in data collection at all stages of implementation [10], as clinician and patient dyads are considered the primary implementation stakeholders in this protocol.

### Quality Improvement Project

Having developed the blueprint, we conducted a small quality improvement project at 2 local CSC clinics to gather feedback that would further inform the design. Two local clinics were included in the project so that we could explore different DMHI configurations ranging from hyperpersonalized to more standardized configurations. Given the quality improvement design of the study, this project did not analyze symptomatic or functional outcomes. Future iterations of iTECSC would benefit from integrating both implementation outcomes and effectiveness outcomes.

## Methods

### Participants

The participants were patients and clinicians at CSC Boston–based clinics for CHR-p care (Center for Early Detection Assessment and Response to Risk [CEDAR]; [17]) and FEP care (Advancing Services for Psychosis Integration and Recovery [ASPIRE]). Patients at CHR-p met broadly defined CHR-p criteria [18], and patients with FEP had at least 1 documented incident of a first episode of psychosis. Clinicians included psychiatrists, a psychologist, a social worker, and a supported education and employment specialist.

### Procedures

At CEDAR, a patient-needs–identifying approach was used when implementing the DMHI into treatment. Patients selected from a “menu” of activities that the DMHI could support including a thought record, mindfulness activities, positive affirmations, relaxation techniques, and psychoeducation materials. Their app was tailored to include their specific “prescription” of activities.

At ASPIRE, a modular approach was presented to patients and clinicians, in which all patients received a preprogrammed app that included surveys measuring mood, sleep, sociality, psychosis, anxiety, and medication that were delivered daily in the morning and evening and as interventions targeting mindfulness, psychoeducational tips, and cognitive restructuring activities.

### Ethical Considerations

The review and analysis of data collected through this quality improvement project was approved by Beth Israel Deaconess Medical Center Institutional Review Board (#2023P000231). Participants were not compensated for participation in this project as it was originally implemented as an augmentation to current care.

### Measures

#### Amended Version of the AACCS Survey

The AACCS survey [11] is a Likert-style survey rated on a 0 to 7 (strongly disagree to strongly agree) scale that assesses the 5 domains of AACCS. The survey was shortened for this study, excluding questions that were not applicable to the project. This survey was administered to both patients and clinicians to identify implementation needs at baseline in both clinics.

#### Evidenced-Based Practice Attitude Scale

The Evidence-Based Practices Attitude Scale (EBPAS [19]) is an 18-item self-report Likert-style survey rated on a scale of 0 to 4 (0=not at all, 1=to a slight extent, 2=to a moderate extent, 3=to a great extent, and 4=to a very great extent) and was used to measure clinician acceptability of new interventions at baseline. The EBPAS measures requirements, openness, appeal, and divergence. High scores in requirements, openness, and appeal demonstrate high levels of acceptability, whereas low scores in divergence suggest high acceptability.

#### Counselor Assessments of Training and Adoption Barriers

The Counselor Assessments of Training and Adoption Barriers [20] survey is a Likert-style survey rated on a scale of 0 to 5 (1=not at all, 2=a little, 3=some, 4=a lot, and 5=very much) that was used at 1-month follow-up to assess the appropriateness of mindLAMP in CSC and was administered to both patients and clinicians.

#### MindLAMP

MindLAMP was used to capture passive and active data to be used for analysis of specific implementation outcomes. MindLAMP was used to assess acceptability (percentage of patients who expressed interest in app or patients who were presented the app), adoption and penetration (percentage of patients and clinicians informed about mindLAMP who used mindLAMP), and feasibility (which patients used what parts of mindLAMP).

#### Post–1-Month Survey

A post–1-month survey was also used to collect data from both patients and clinicians regarding feasibility, fidelity, and sustainability.

### Analyses

Demographic information was analyzed to examine sample diversity. Descriptive statistics, including means and frequencies, was used to examine the baseline and 1-month survey data to assess implementation outcomes. Survey data were qualitatively reviewed to assess for feedback that may provide insights into improving future implementation of projects. Descriptive statistics from the mindLAMP app and clinical data was also analyzed to examine which specific parts of the app had the highest participation to inform future development. One provider from each clinic provided case examples that were analyzed to demonstrate app use over the 1-month period.

## Results

### Demographics and Data Completion

The initial presentation included 4 clinicians from the ASPIRE clinic and 5 clinicians from the CEDAR clinic. Two clinicians from the ASPIRE clinic—JT (MD) and MK (MD)—and 3 clinicians from the CEDAR clinic—Caroline Howland (licensed independent clinical social worker), Amanda Weber (PhD), and JG (BA; supported education and employment specialist)—expressed interest in the DMHI opportunity. Moreover, 1 clinician from ASPIRE (MK) and 3 clinicians from CEDAR (Caroline Howland, Amanda Weber, and JG) completed the baseline assessment battery. Furthermore, 2 ASPIRE clinicians (JT and MK) and 2 CEDAR clinicians (Caroline Howland and JG) worked with patients with the DMHI, and 1 ASPIRE clinician (MK) and 1 CEDAR clinician (JG) completed the follow-up assessment battery. A case study was provided from 1 clinician at each site to describe their clinical use of the DMHI (JT and JG).

The project included 5 participant dyads (CEDAR: n=2 and ASPIRE: n=3). Patients were 40% (2/5) Black or African American and 60% (3/5) White; 40% (2/5) of the participants identified as cis-male, 40% (2/5) of the participants identified as cis-female, and 20% (1/5) of the participants identified as transmasculine; the participants had a mean age of 19.6 years. Moreover, 20% (1/5) of the participants were in high school, 60% (3/5) of the participants attended university, and 60% (3/5) of the participants were employed. Furthermore, 3 patients completed the baseline and follow-up assessment batteries.

### AACCS Quantitative Survey

The AACCS quantitative survey data (Table 1) demonstrated that clinicians and patients at both clinics on average agreed that they had high levels of access to technology, felt connected to the technology, and believed that technology could be sustained over time, as responses to the AACCS survey for all categories ranged from 5 (somewhat agree) to 6 (agree).

**Table 1:** Access, Alignment, Connection, Care, and Scalability (AACCS) responses and implementation outcomes for patients and clinicians at Center for Early Detection Assessment and Response to Risk (CEDAR) and Advancing Services for Psychosis Integration and Recovery (ASPIRE).

			CEDAR				ASPIRE					Total
			Patients		Clinicians		Patients		Clinicians			
**AACCS, mean (SD; range)**												
	Access	5.66 (0.5; 1-7)		6.33 (0.94; 1-7)		6.66 (0.47; 1-7)		6 (0; 1-7)		6.16 (0.36; 1-7)		
	Connection	5.25 (1.25; 1-7)		5.66 (1.41; 1-7)		6.5 (0.5; 1-7)		5.33 (0.57; 1-7)		5.69 (0.49; 1-7)		
	Sustainability	6.5 (0.5; 1-7)		6.33 (0.47; 1-7)		6 (0; 1-7)		5 (0; 1-7)		5.96 (0.58; 1-7)		
**Acceptability^a^, mean (SD; range)**												
	Requirements	—^b^		2.67 (1.25; 0-4)		—		3 (0; 0-4)		2.85 (1.09; 0-4)		
	Appeal	—		2.67 (0.24; 0-4)		—		2.75 (0.43; 0-4)		2.71 (0.21; 0-4)		
	Openness	—		2.83 (0.24; 0-4)		—		2.25 (0.43; 0-4)		2.54 (0.32; 0-4)		
	Divergence	—		0.92 (0.31; 0-4)		—		2 (0; 0-4)		1.46 (0.54; 0-4)		
Adoption or penetration (percentage of patients or clinicians informed about the app who used it), n/N (%)			2/5 (40)		2/5 (40)		3/3 (100)		2/4 (50)		9/17 (53)	
Appropriateness^c^, mean (SD; range)			3.16 (0.69; 1-5)		3.5 (0.5; 1-5)		—		3.66 (0.47; 1-5)		3.32 (0.6; 1-5)	
Sustainability (post–1-month survey, “Will you continue to use as part of treatment?”), n/N (%)			2/2 (100)		1/2 (50)		0/1 (0)		1/1 (100)		4/6 (67)	

^a^Acceptability measured by the Evidence-Based Practice Attitude Scale.

^b^Not available.

^c^Appropriateness measured by Counselor Assessments of Training and Adoption Barriers.

In assessing how technology could align with care offered by the clinics, clinicians rated supporting psychotherapy as the highest, but overall responses were diverse, as clinicians reported that the DMHI could support unique therapeutic interventions addressing specific treatment needs and all aspects of FEP or CHR-p care. This is best illustrated in the cases presented in Textbox 1.

**Clinical interventions identified by clinicians and patients to be used in the app.**

**Center for Early Detection Assessment and Response to Risk (CEDAR) patients**

- Journaling
- Feeling tracking
- Case management
- Organizational skills
- Executive functioning
- Relaxation
- Daily ritual to center self

**CEDAR clinicians**

- Mindfulness for acceptance and commitment therapy–based therapy
- Psychoeducation for mood disorders
- Medication tracking
- Worksheets for cognitive behavioral therapy type therapy
- Exposure type work
- Daily structure building
- Coping skills training
- Tracking thoughts, feelings, and behaviors
- Out-of-session work reminders
- Treatment progress tracking
- Coping skills practice and education

**Advancing Services for Psychosis Integration and Recovery (ASPIRE) patients**

- Establish routine
- Tracking thoughts
- Practice mindfulness

**ASPIRE clinicians**

- Measurement-based care

### Baseline Implementation Outcomes

**Figure 6:**
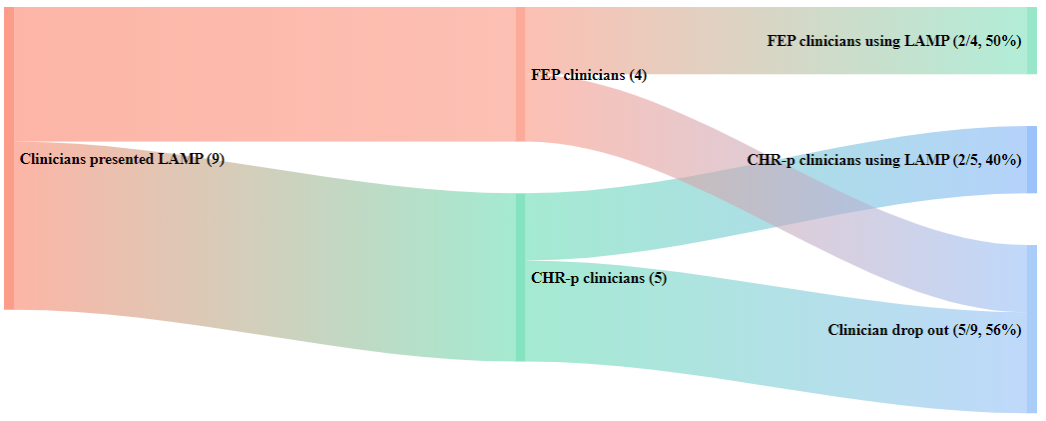
Implementation pathways for first-episode psychosis (FEP) and clinical high risk for psychosis (CHR-p) clinicians.

**Figure 7:**
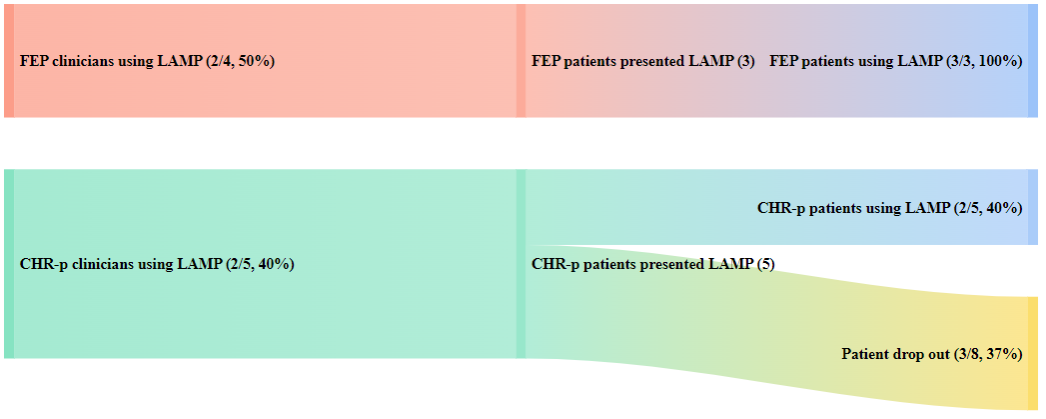
Implementation pathways for first-episode psychosis (FEP) and clinical high risk for psychosis (CHR-p) patients.

Implementation outcomes data suggested that patients and clinicians at both clinics had moderately high levels of acceptability, reflected by the moderately high mean scores from the EBPAS ranging from 2 (moderate extent) to 3 (great extent) for adherence to requirements, appeal, openness, and low levels of divergence to new interventions (Table 1). Regarding adoption and penetration, 40% (2/5) of the patients and 40% (2/5) of the clinicians at CEDAR participated in the intervention, whereas 100% (3/3) of the patients and 50% (2/4) of the clinicians at ASPIRE participated in the intervention. The total level of adoption and penetration at both clinics for both patients and clinicians was 53% (9/17), suggesting moderate levels of adoption and penetration, slightly favoring the modular version of the app for patients that was presented to ASPIRE. The implementation pathways from the initial presentation to adoption and penetration outcomes are shown in Figures 6 and 7.

### Post–1-Month Outcomes

The Counselor Assessments of Training and Adoption Barriers survey responses that were collected during the 1-month follow-up suggested that on average clinicians and patients rated the app with *some* (3) appropriateness to *a lot* (4) of appropriateness. Post–1-month follow-up survey responses demonstrated that all 3 patients preferred different aspects of the app (Table 2). These results also suggested that patients had varying difficulties and challenges with the app, ranging from being unsure of what to do outside the scheduled activities, using the technical aspects of the app, and reminding themselves to use the app. These data demonstrate moderate feasibility, given the wide range of needs and challenges while using the DMHI.

**Table 2:** Post–1-month survey responses.

Clinic and participant		“What was your favorite part of the app?”	“Did you have any challenges with the app?”	“Will you continue using as part of treatment?”	“What do you wish the app could also do?”	“Do you have any other feedback for us about the app?”
**ASPIRE^a^**						
	Patient 1	“The daily recaps”	“Figuring out what to do outside of the daily tasks”	“Possibly”	“Nothing I am satisfied with the app”	“I may not use it as much moving forward just because of lack of time”
	Clinician 1	“Patient Reports that the app was easy to use”	“No”	“Yes”	“Any Biosensor Information (heart rate, HRV, Sleep)”	“None”
**CEDAR^b^**						
	Patient 1	“The morning affirmations.”	“Using the app is just fine however reminding myself to track my use once I’m done has been a struggle.”	“Yes I will try my best to use it.”	“I wish I could put in specific times for my activities and then have the app remind me at that specific time for each activity.”	“None at the moment. I would wish the breathing exercise is a bit longer.”
	Patient 2	“My favorite part of the app was seeing the progress you made, but seeing the dots graph of seeing previous answers to track goals/activities, and see what I answered before”	“Remembering how to use it was difficult. Flipping through/looking through different parts of it, but I got in the habit of knowing where things are.”	“Yes!”	“It would be really cool if part of the journaling you could add photos/zines, kind of like in an Instagram fashion that you could put words under to help remember things more clearly.”	“I think it would also be cool if you could customize it to make the app pink for yourself—to make it fun!”
	Clinician 1	“I really enjoyed being able build the app based on my client’s needs, and that the client and I could collaborate together to structure the modules in the app.”	“It was difficult to engage the client in using the app as we had originally intended.”	“Yes”	“I wish the app may also have some structured modules that could be used in a systemic way, clients might also have an easier time signing on to a predesigned program.”	“No”

^a^ASPIRE: Advancing Services for Psychosis Integration and Recovery.

^b^CEDAR: Center for Early Detection Assessment and Response to Risk.

### Case Examples

Deidentified case examples are provided from 1 provider from each clinic to describe their experiences with mindLAMP (Textbox 2). These case examples demonstrate how DMHIs can be deployed to address the unique needs of each patient.

**Deidentified case examples.**

**Advancing Services for Psychosis Integration and Recovery (first-episode psychosis)**

- Steve is a male with early course psychosis who sought treatment for worsening delusions and hallucinations. He initially used the app to help track when he had hallucinations and assess the impact of different dosages of medications on his delusions and hallucinations. In using the app he discovered cognitive assessments related to trails-A/B tests and found that engaging in these tests provided distraction that also helped to reduce his voices. He tried relaxation exercises in the app but did not find them helpful. With more stability in his symptoms, he next used the app for behavioral activation and goal setting. He found the app helped keep him accountable to his therapist and was able to begin to take longer and more frequent walks. He enjoyed being able to share his progress in sessions. After about three months he felt that he no longer needed to use the app as he understood the impact of his medication. He had internalized new skills to cope with hallucinations/delusions and was able to goal set without the aid of the app.

**Center for Early Detection Assessment and Response to Risk (clinical high risk for psychosis)**

- Summer is an 18-year-old transgender male high-school student experiencing psychosis risk symptoms including worsening perceptual abnormalities, unusual thought content, and disorganized thinking. He sought treatment to prevent conversion to a first episode of psychosis, to improve social and vocational functioning, and to improve emotional regulation skills. He participated in the menu-style approach to using the app and asked for the app to be built to assist in supporting organizational skills, tracking emotions, reducing stress, and providing mindfulness activities. He found the app was helpful in being able to track different emotions over time. He also found the meditation activities useful in reducing stress, and the daily structure builders to be helpful in improving organizational skills through providing reminders and a structure to plan his day. He reported that while the journaling reminder was helpful, he did not feel comfortable journaling directly into the app and preferred to use the app as a reminder tool to journal in a notebook that was more private. After the course of 1 month, he reported he would like to continue to use the app in concert with regular treatment.

## Discussion

### Principal Findings

The results of this quality improvement study shed light on the current barriers and possible facilitators for the implementation of DMHIs in CSC. Primarily, this quality improvement project demonstrated that clinicians and patients reported high levels of access to DMHIs and believed in the sustainability of DMHIs. In addition, the results suggest that clinicians and patients express an interest in a diverse range of use cases for DMHI-augmented care. Furthermore, patients and clinicians self-reported moderate to high levels of acceptability on the AACCS survey, demonstrating initial acceptability and interest in DMHIs generally. When implemented into care, adoption and penetration proportions ranged from 40% to 100% in each setting, with an average of 53%, suggesting moderate and variable adoption and penetration across different use cases. At 1-month follow-up, clinicians and patients reported *some* to *a lot* of appropriateness of DMHIs in CSC care after using the intervention, suggesting that the DMHI was moderately appropriate for use in these care settings. Post–1-month follow-up survey results further suggest variable feasibility of the DMHI, given the diversity of use cases and challenges that arose while using the DMHI. Finally, the case examples demonstrated how and when DMHIs can be used and designed uniquely so that they can address the specific needs of each patient to inform care. This suggests that flexibility and versatility are important when considering the use of DMHIs in care. These results indicate that barriers to implementation are exemplified during the adoption and penetration phase of implementation, which may be associated with the moderate feasibility and appropriateness of the DMHI in its presented form. To improve implementation outcomes of DMHIs in CSC, barriers and facilitators to adoption and penetration, feasibility, and appropriateness must be investigated and targeted.

When reviewing these findings, it is evident that both clinicians and patients can access and believe in the sustainability of DMHIs, demonstrating their readiness to enhance care with DMHIs, which is reflective of mainstream findings in the literature [3].

While considering what may have facilitated higher levels of adoption and feasibility, this project suggested that the versality and multicomponent design, rather than the complexity, of DMHIs is important as evidenced by the diversity of responses to how clinicians and patients imagined the DMHI could be used to align with treatment goals. This is reflective of similar findings, which found that approaching DMHI development from a versatile rather than a complex perspective may be preferable [21]. Results from the case examples and follow-up survey responses also suggest that DMHI autonomy is important to allow the DMHI to appropriately align and augment the unique clinical styles of clinicians and the needs of patients, which has also been evident in other recent findings [22].

Congruent with other trends in the literature [23], patient feedback and higher rates of fidelity and feasibility in the modular version of the DMHI suggest that a structured approach may maximize acceptability, adoption, appropriateness, fidelity, and feasibility. In addition, findings from a recent qualitative study gathering clinician feedback on another DMHI suggested that adaptable DMHIs may work to improve implementation outcomes such as feasibility, appropriateness, adoption, and penetration [24]. Prior research using smartphone apps in the care of patients with early psychosis has also shown feasibility [9,25], which is similar to our results; however, the use of digital navigators in this study is novel. The implementation methodology described in this project heavily relied upon digital navigators, thus reinforcing the essential role of a digital navigator in the initial and sustained implementation of DMHIs in clinical settings [15].

Given the findings from the quality improvement project as well as leading trends in mainstream literature, we suggest that developing DMHIs that are versatile rather than complex in their design, promote patient and clinician autonomy to meet unique clinical needs, and provide patients with a structure to complete modules and activities may improve the evident implementation pitfalls of DMHIs exemplified in the adoption, penetration, appropriateness, and feasibility outcomes.

Although this quality improvement project was limited by its protocol design, sample size, and absence of symptomatic or functional outcome data, a recent study implementing a DMHI into an FEP program showed promise in reducing relapses, hospitalization, and visits to urgent care units [25]. Our results suggest that the greatest barriers to adoption and penetration may be associated with feasibility and appropriateness. Developing DMHIs that are adaptable, versatile, structured, and patient- and clinician-centered may improve feasibility and appropriateness, thus improving adoption and penetration outcomes, which may in turn allow for patients to access the identified benefits that technology-supported CSC has to offer.

### Limitations

This quality improvement project was limited by many factors, as it was conducted as natural quality improvement project in real-world clinical settings. Our primary limitation included a small sample size, given our goal to establish feasibility. In addition, the authors recognize that some of the findings may be biased, given that the authors of the protocol design have participated in the project as clinicians; however, many of the suggestions that had been leveraged to improve the protocol came from patients and clinicians who were not directly involved in the development of the protocol. In addition, this study did not assess symptomatic or functional outcomes. Future iterations of iTECSC may benefit from assessing implementation outcomes and effectiveness outcomes as primary co-outcomes.

### Conclusions

In summary, the feasibility findings from this project have informed the development of the iTECSC protocol by suggesting the need for a patient-centered approach that is guided by digital navigators and provides versatility, autonomy, and structure. Such a new study protocol has the potential to build on growing insights regarding the need for versatility, autonomy, digital navigator support, and structured applications [26-28]. We anticipate that by continuing to research and improve implementation barriers impeding the adoption and penetration of DMHIs in CSC, accessibility and uptake of DMHIs in CSC will improve, thus connecting patients to the demonstrated benefits of technology-augmented care [25].

## Abbreviations

AACCS: Access, Alignment, Connection, Care, and Scalability
ASPIRE: Advancing Services for Psychosis Integration and Recovery
CEDAR: Center for Early Detection Assessment and Response to Risk
CHR-p: clinical high risk for psychosis
CSC: coordinated specialty care
DMHI: digital mental health intervention
EBPAS: Evidence-Based Practices Attitude Scale
FEP: first-episode psychosis
iTECSC: Implementing Technologies to Enhance Coordinated Specialty Care

## Data Availability

The data have not been published as it is naturalistic data collected in a real-world setting. Anonymized data may be shared upon request to the author.
